# Calabar Bean

**Published:** 1869-07-01

**Authors:** 


***Calabar Bean.***

Dr. William Wright communicates to the Canada *Med. Journal* a valuable paper upon this agent, asserting it to be locally an antidote to belladonna, and generally to strychnia, and as a remedy in tetanus. Its efficacy in this latter instance, however, is limited by the degree of lesion of the peripheral nerves, the agent haviug no control over the afferent or sensory nerves, and does not suspend their conductivity.

It would seem from the above that the calabar bean occupies a similar relation to tetanus as does chloroform, simply suspending the reflex irritability of the spinal cord, but as not moderating the irritation of the peripheral extremities of the nerves. As this is, in the vast majority of cases, the pathognomonic element in tetanus, we fear that the treatment of this formidable malady is "*adhuc subjudice.*"

